# Association of cardiorespiratory fitness with phenotypic age in younger population: a study based on the NHANES database

**DOI:** 10.3389/fspor.2025.1503135

**Published:** 2025-07-10

**Authors:** Yi Shen, Wanying Shen, Yongyao Shen, Bo Chen, Cui Wu, Liying Jiang

**Affiliations:** ^1^ Department of Epidemiology, School of Public Health, Nantong University, Nantong, Jiangsu, China; ^2^ Department of Epidemiology, College of Public Health, Shanghai University of Traditional Chinese Medicine, Shanghai, China; ^3^ Department of Non-Communicable Disease, Baoshan District Center for Disease Control and Prevention in Shanghai, Shanghai, China; ^4^ Jiading Central Hospital, Shanghai University of Medicine & Health Sciences, Shanghai, China; ^5^ Department of Prevention Medicine, College of Public Health, Shanghai University of Medicine & Health Sciences, Shanghai, China

**Keywords:** cardiorespiratory fitness, phenotypic age, obesity, NHANES, healthy aging

## Abstract

**Background:**

Phenotypic age (PA), a novel signature of morbidity and mortality risk based on clinically collected parameters, is considered one of the most promising biomarkers for capturing aging. However, unequivocal evidence on the link between cardiorespiratory fitness (CRF), assessed by estimated maximal oxygen consumption (Vo_2max_), and PA remains scarce, particularly within the first half of life. This study aims to explore the relationships between CRF and the age-adjusted value derived from the residuals of the regression of PA on chronological age (PhenoageAcceleration: PAA), uncovering the prognostic value of CRF in the early lifetime to provide perspectives for understanding and improving healthy aging.

**Methods:**

Data from 3,069 participants in the National Health and Nutrition Examination Survey (NHANES) were included and further examined. CRF status was determined by Vo_2max_ according to gender and age-specific criteria, with low and moderate levels classified as impaired CRF. PA was calculated from multisystem blood biomarkers and chronological age. The association of CRF status with cross-sectional PAA was investigated, and subgroup analyses were further performed to explore and identify potentially vulnerable populations.

**Results:**

In the multivariable logistical regression analysis, maintenance of CRF was significantly and inversely associated with PAA, demonstrating a decreased risk of 42% in the high CRF group [OR (95% CI): 0.58 (0.36, 0.96), *p* = 0.033]. Compared with those with non-impaired CRF, those in the impaired group exhibited a rise in PA by 1.46 years [*β* (95% CI): 1.46 (1.03, 2.10), *p* = 0.040]. Interestingly, in the population of over 29 years’ old, a significant interaction between obesity and impaired CRF for PAA was observed (*p* = 0.018; *p* = 0.026).

**Conclusions:**

Poor CRF may serve as a potential risk factor for accelerated biological aging (BA) in relatively young populations, and the existence of obesity could exacerbate the aging process. This represents a potential intervention target for promoting healthy aging across different age groups in the future.

## 1 Introduction

Population aging, attributed in part to the global transition from high to low mortality rates, has seen life expectancy surge over twofold since 1900 (1, 2). However, this longevity gain masks a critical distinction: while life expectancy quantifies survival duration, healthy life expectancy—defined as years lived without major disability—reflects the quality of an extended lifespan. In fact, there is a substantial gap of approximately 10 years between the two, irrespective of countries regionally and financially (3). The paradox of surviving longer yet spending more years in ill-health not only erodes individual living quality but also amplifies socioeconomic losses through escalated medical expenditures and long-term caregiving burdens. To improve this situation and extend the health span, strategies that target aging itself to ease and even reverse the physiological deterioration associated with it have garnered widespread interest across interdisciplinary fields, given its role as the strongest risk factor for multiple morbidities, ranging from cardiovascular ailments to neurological disorders (4). While chronological age (CA) typically serves as a measure of aging, the great heterogeneity in age-related pathologies risk among individuals of the same age suggests variations in aging rate within their intrinsic biological processes (5). Researchers have been striving for years to develop a systematic framework to consolidate the extensive biological effects of the aging process, with the implicit pursuit of devising a quantitative, analytical, and predictive approach to understand healthy aging.

Several methods for measuring biological aging (BA) have emerged as the times require, encompassing telomere length, deficit-accumulation frailty index, epigenetic clocks derived from DNA methylation markers, and algorithms that integrate information from standard clinical parameters (6–9). The last method among these, which utilizes machine learning algorithms to amalgamate information from composite blood chemistries routinely collected into a single latent variable, has been considered one of the most promising tools for BA assessment (10). While ensuring measurement accuracy, it is more cost-effective compared with other measures of BA, making it feasible for large-scale studies. Phenotypic age is a previously published and overwhelmingly validated biological-age algorithm based on nine clinical parameters and may represent the manifestations of varied hallmarks of aging occurring at multiple dimensions of cells, tissues, and organs, providing a more holistic profile of aging dynamics of the whole organism (11). It is more practical for researchers to be adept at capturing the trajectory of vulnerability to injury, illness, and death across diverse subpopulations on time scales (12).

Cardiorespiratory fitness (CRF), typically expressed as maximal oxygen consumption (Vo_2max_) measured through non-exercise algorithms and exercise-based tests, is indicative of the capacity of bodily working tissues to absorb, transport, and utilize oxygen during sustained vigorous activity that involves large muscle mass to reflect actual physical fitness, setting itself apart from the physical activity evaluated by self-report questionnaires (13) Since the mid-20th century, retrospective studies from major cohorts with tracking periods ranging from 6 to 28 years have established a graded inverse relationship between CRF and both disease-specific and all-cause mortality. Key evidence includes, but is not limited to, the Aerobics Center Longitudinal Study (ACLS, USA; *n* = 14,355, predominantly non-Hispanic White males, mean age 44 years), which demonstrated a 15% reduction in all-cause mortality per 1-MET improvement in CRF; the Veterans Affairs Study (VA, USA; *n* = 15,660, non-Hispanic Black and White males, mean age 59 years) found a 13% risk decline per 1-MET. Complementing these findings, the Henry Ford Exercise Testing (FIT) Project further confirmed the mortality-predictive capacity of CRF across diverse populations (14–16). This underlies that this robust relationship might imply a broader health significance, i.e., optimal CRF may mitigate the loss of the health span, beyond simply lifespan, in humans. Research linking CRF to BA could lend credibility to this point, yet evidence from population-based studies is still limited. A positive and significant relationship between CRF and telomere length, mainly among the middle-aged and elderly, seems to exist in those past works (17). More recently, Kawamura and colleagues conducted a cross-sectional study involving 144 older men to explore the relationship between CRF and DNAm aging clocks, revealing that maintenance of CRF is associated with delayed BA (18). Further investigation is warranted to deepen the relationship between CRF and BA assessed with the most clinically relevant approaches. Moreover, it is noteworthy that such links in younger populations, who represent a highly attractive target for interventions aimed at extending the health span, remain uncovered. There is an urgent need for literature to support the prognostic value of CRF in early lifetime, thus actively responding to global healthy aging.

Therefore, we sought to shift from mortality to BA as an investigation focus when a study tends to have CRF discussed by determining the association between the altered CRF status and changes in Phenoage (PA) concurrently among the younger population. This could serve as one part of the natural process in understanding the multifaceted role of CRF in the promotion of healthy aging.

## 2 Materials and methods

### 2.1 Study design and population

The National Health and Nutrition Examination Survey (NHANES) is a biennial survey that selects a group of children and adults being representative of non-institutionalized US population to collect information on health and nutrition through a complex, multistage, probability sampling design. The NHANES study pilot was conducted with the approval of the ethics review committee of the National Center for Health Statistics (NCHS). Written informed consents were obtained from all participants. For more information on NHANES data access and description, please visit the following address: https://www.cdc.gov/nchs/nhanes/index.htm.

**Figure 1:**
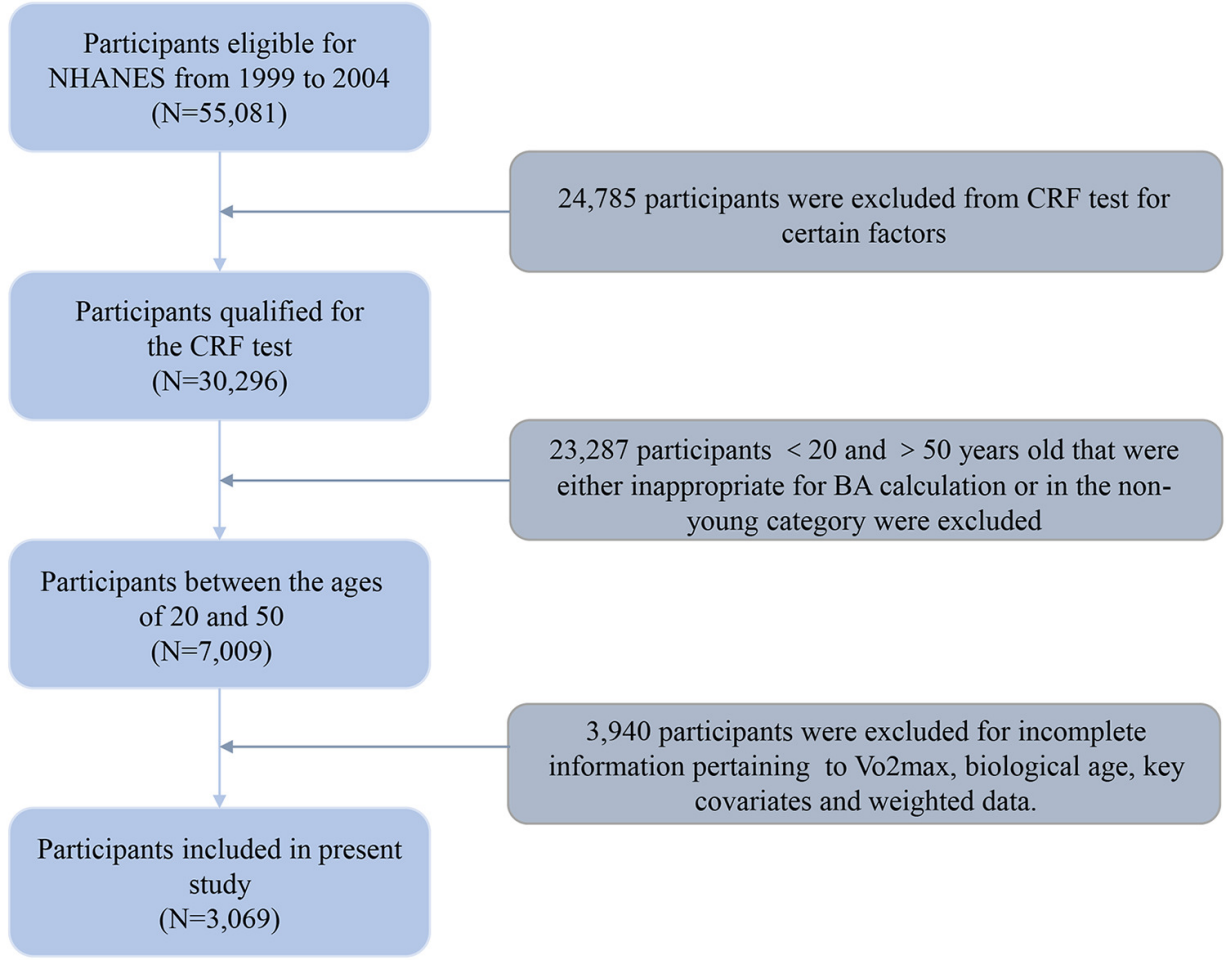
Flowchart of the participant inclusion process.

Data from three survey waves (1999–2000, 2001–2002, and 2003–2004) of the NHANES were drawn and merged, and a total of 55,081 individuals were initially enrolled in our study. However, 24,785 individuals were excluded as they were ineligible for the CRF test due to factors such as pregnancy, physical functioning limitations, cardiovascular diseases/symptoms, lung/breathing conditions/symptoms, asthma symptoms, uncertain medication, or other reasons rendering them unfit for such an examination. Of these, 23,287 participants aged under 20 and over 50 years old, being either inappropriate for BA calculation or in the non-young category, were also excluded. Also, 3,940 individuals lacking sufficient information for calculating Vo_2max_, biological age, as well as key covariates were further excluded from the analytic sample, resulting in 3,069 participants selected for the final dataset. The detailed exclusion criteria and study flow chart are shown in Figure 1.

### 2.2 Assessment of CRF

The CRF assessment was performed by skilled healthcare technicians. Participants were allocated to one of eight treadmill test protocols according to their gender, age, body mass index (BMI), and self-reported physical activity levels, and outcomes of the test were used to evaluate maximum oxygen uptake (Vo_2max_). The whole procedure for estimating Vo_2max_ is elaborated upon in the Supplementary file 1. The cut point for gender- and age-specific CRF levels among adults aged 20–49 was determined using data from the Aerobics Center Longitudinal Study (ACLS), with three categories of adjusted levels classified as low, moderate, and high. This approach accounts for physiological differences in Vo_2max_ between genders and across age groups, ensuring comparable fitness thresholds within each demographic subgroup. For more details, please refer to the Supplementary file 2. Specifically, low and moderate CRF levels were considered impaired CRF in this study (19, 20).

### 2.3 Assessment of signs of BA

Assessment of BA in this study is one of the widely accepted algorithms introduced by Levine et al. in 2018, known as the phenotypic age (11). It was originally constructed by regressing the hazard of age-related specific mortality, including mortality from heart disease, cerebrovascular disease, diabetes mellitus, chronic respiratory disease, malignant neoplasms, Alzheimer's disease, etc., on nine blood biomarkers and chronological age data with the parametrization of Gompertz proportional hazard models. The output of the algorithm estimates a “mortality score,” which is then converted into a value of biological age, namely PA in this context, and corresponds to the chronological age at which a participant's mortality risk in the NHANES III reference population would be approximately normal. The specific calculation formulae of the phenotypic age algorithm metrics are as follows:

$$\mathrm{Phenoage}=141.50225+\frac{\ln\;[-0.0053\times \ln\;(1-\mathrm{Mortality}\;\mathrm{Risk})]}{0.090165}$$

$$\mathrm{Mortality}\;\mathrm{Risk}=1-\exp\left(\frac{-1.51714\times \exp(xb)}{0.0076927}\right)$$

$$\begin{array}{rl} xb & =-19.907-0.0036\times \mathrm{albumin}+0.095\times \mathrm{creatinine}+0.1953\\ & \;+\times \mathrm{glucose}0.0954\times \ln\;(C-\mathrm{reactive}\;\mathrm{protein})-0.0120\\ & \;+\times \mathrm{lymphocyte}\;\mathrm{percentage}0.0268\times \mathrm{mean}\;\mathrm{corpuscular}\;\mathrm{volume}\;\\ & \;+0.3306\times \mathrm{red}\;\mathrm{blood}\;\mathrm{cell}\;\mathrm{distribution}\;\mathrm{width}+0.00188\;\\ & \;\times \mathrm{alkaline}\;\mathrm{phosphatase}+0.0554\times \mathrm{white}\;\mathrm{blood}\;\mathrm{cell}\;\mathrm{count}\\ & \;+0.0804\times \mathrm{chronological}\;\mathrm{age}. \end{array}$$

The corresponding code could be acquainted in the R package (https://github.com/dayoonkwon/BioAge).

A natural logarithmic transformation was performed for biomarkers that are not normally distributed before commencing the calculation of BA measurement. The correlation coefficient between the constructed biological age founded on the aforementioned biomarkers and CA was 0.97 in our study. In addition, the PhneoAgeAccel (PAA), determined by the residual between biological age estimated from PA against chronological age that is greater than zero, is indicative of the acceleration of BA, that is, a person appears older than expected, physiologically. A positive PAA suggests a higher risk for disability, and mortality, and vice versa.

### 2.4 Definition of covariates

Given the potential confounders in previous studies affecting CRF or BA, a constellation of factors from this framework was included and adjusted to ensure a robust analysis, as follows: age, gender, ethnicity, education background, poverty income ratio (PIR), marital status, cigarette smoking, alcohol consuming, physical activity, obesity, hypertension, and diabetes. Please see detailed definitions and grouping for covariates in Supplementary file 3.

### 2.5 Statistical analysis

Weights were considered in all statistical analyses in accordance with the NHANES guidelines to provide nationally representative estimates (WTMEC4YR × 2/3+ WTMEC2YR × 1/3).

As descriptive data, continuous variables not normally distributed were presented as median (Q1, Q3), and categorical variables were presented as counts (%). The difference of continuous and categorical variables between CRF groups was evaluated by means of the Mann–Whitney *U* test and chi-square test, respectively. Weighted multivariable logistic regression equations were employed to explore the relationship between the CRF status and PAA, with the estimated odds ratios (ORs) and 95% confidence intervals (95% CIs) being presented accordingly, and *p* for trend was also calculated. The sequence of three discrete modeling strategies for logistic analysis proceeded as follows: Model Ⅰ was a crude model adjusted for no covariate. Apart from essential demographic characteristics (age, gender, and ethnicity), Model Ⅱ was further adjusted for socioeconomic status (education attainment, PIR, and marital status). Model Ⅲ, the fully adjusted model considering a myriad of potential influences, expanded the adjustment scope of Model Ⅱ to encompass a broader range of covariates (cigarette smoking, alcohol consuming, physical activity, obesity, hypertension, and diabetes). The covariates examined in each model were listed in the footnotes of the corresponding tables. In order to explore potential effect modifiers, we conducted a subgroup analysis based on gender, ethnicity, smoke, and obesity status. In addition, the population was stratified by predefined age groups for further subgroup analysis. All statistical analyses for this study were carried out using R version 4.2.3, and a two-sided *p* < 0.05 was considered significant.

## 3 Results

### 3.1 Baseline characteristics

A total of 3,069 participants were assembled, from whom it was extrapolated to mirror an estimated weighted population of 7.91 million in the United States. Table 1 presents the detailed baseline characteristics of our participants in the final analysis. Briefly, the distribution of subjects by gender and age group was relatively balanced. The majority of participants were non-Hispanic White (72%), and approximately one-quarter were current smokers, and one-third were heavy drinkers. There was a certain prevalence of hypertension (13%) and diabetes (3.5%) within the population.

**Table 1:** Characteristics of study participants.

Characteristic	Overall, *N* = 3,069 (100%)^a^
Age (%)	
20–29	1,149 (33%)
30–39	1,048 (36%)
40–49	872 (30%)
Gender (%)	
Male	1,640 (52%)
Female	1,429 (48%)
Race (%)	
Non-Hispanic White	1,458 (72%)
Mexican American	779 (8.5%)
Non-Hispanic Black	591 (9.6%)
Other	241 (10.0%)
Married with partner (%)	
No	1,197 (38%)
Yes	1,770 (62%)
Education level (%)	
Below high school	678 (14%)
High School or above	2,391 (86%)
PIR (%)	
Not poor	2,184 (83%)
Poor	673 (17%)
Smoking (%)	
Never	1,759 (57%)
Former	484 (17%)
Current	826 (26%)
Drinking (%)	
Former	300 (9.7%)
Heavy	891 (29%)
Mild	910 (33%)
Moderate	527 (18%)
Never	319 (10%)
Physical activity (%)	
Inactive	1,578 (66%)
Active	847 (34%)
Obesity (%)	
No	2,242 (77%)
Yes	821 (23%)
Hypertension (%)	
No	2.632 (87%)
Yes	400 (13%)
Diabetes (%)	
No	1,454 (97%)
Yes	82 (3.5%)
Vo_2max_ ml/kg/min	40.53 ± 9.6
CRF status	
Low	506 (14%)
Moderate	1,055 (34%)
High	1,508 (52%)
Impaired CRF	
No	1,508 (52%)
Yes	1,561 (48%)
PA	29 ± 9
PAA	
No	330 (8.9%)
Yes	2,739 (91%)

^a^ Median (SD) for continuous; *n* (%) for categorical.

PIR, ratio of family income to poverty; CRF, cardiorespiratory fitness; PA, phenoage; PAA, phneoageaccel.

We summarized baseline information of participants on the basis of CRF levels in Table 2. Of those, subjects in the low CRF group consisted of more young females, Mexican Americans, and non-Hispanic Blacks, exhibiting higher tendencies for poverty, obesity, hypertension, and diabetes compared with those in the high CRF group.

**Table 2:** Baseline characteristics of the study participants according to the levels of the CRF.

Characteristic	Low, *N* = 506 (14%)^a^	Moderate, *N* = 1,055 (34%)^a^	High, *N* = 1,508 (52%)^a^	*p-v*alue^b^
Age (%)				0.001
20–29	224 (42%)	400 (35%)	525 (30%)	
30–39	185 (41%)	364 (37%)	499 (35%)	
40–49	97 (17%)	291 (29%)	484 (35%)	
Gender (%)				0.021
Male	238 (44%)	577 (52%)	825 (54%)	
Female	268 (56%)	478 (48%)	683 (46%)	
Race (%)				0.002
Non-Hispanic White	188 (60%)	505 (73%)	765 (75%)	
Mexican American	138 (10%)	246 (7.6%)	395 (8.5%)	
Non-Hispanic Black	134 (16%)	215 (9.6%)	242 (8.0%)	
Other	46 (14%)	89 (9.9%)	106 (8.9%)	
Married with partner (%)				0.72
No	192 (38%)	396 (36%)	609 (39%)	
Yes	295 (62%)	632 (64%)	843 (61%)	
Education level (%)				0.24
Below high school	114 (17%)	208 (13%)	356 (15%)	
High school or above	392 (83%)	847 (87%)	1,152 (85%)	
PIR (%)				0.031
Not poor	355 (78%)	758 (82%)	1,071 (85%)	
Poor	118 (22%)	226 (18%)	329 (15%)	
Smoking (%)				0.052
Never	836 (55%)	318 (62%)	605 (58%)	
Former	248 (18%)	70 (16%)	166 (16%)	
Current	424 (26%)	118 (22%)	284 (27%)	
Drinking (%)				0.54
Former	55 (13%)	108 (10%)	137 (8.4%)	
Heavy	147 (28%)	297 (28%)	447 (29%)	
Mild	145 (32%)	321 (32%)	444 (34%)	
Moderate	71 (15%)	178 (18%)	278 (19%)	
Never	67 (12%)	110 (11%)	142 (9.0%)	
Physical activity (%)				0.25
Inactive	232 (65%)	566 (66%)	780 (66%)	
Active	133 (35%)	282 (34%)	432 (34%)	
Obesity (%)				<0.001
No	293 (62%)	776 (78%)	1,173 (81%)	
Yes	212 (38%)	276 (22%)	333 (19%)	
Hypertension (%)				0.005
No	412 (81%)	901 (88%)	1,319 (89%)	
Yes	85 (19%)	144 (12%)	171 (11%)	
Diabetes (%)				<0.001
No	234 (91%)	473 (98%)	747 (97%)	
Yes	24 (9.4%)	18 (2.0%)	40 (2.7%)	
Vo_2max_ ml/kg/min	29.1 ± 4.2	36.2 ± 4.5	47.2 ± 9.7	<0.001
PA	28.2 ± 9.1	29.2 ± 9.4	29.1 ± 9.3	0.090
PAA				0.006
No	416 (86%)	948 (91%)	1,375 (92%)	
Yes	90 (14%)	107 (8.7%)	133 (7.6%)	

^a^ Median (SD) for continuous; *n* (%) for categorical.

^b^ Chi-squared test with Rao & Scott's second-order correction; Wilcoxon rank-sum test for complex survey samples.

CRF, cardiorespiratory fitness; PIR, ratio of family income to poverty; PA, phenoage; PAA, phneoageaccel.

### 3.2 Association between CRF and BA

Compared with individuals with low CRF, participants maintaining high levels exhibited a lower risk of accelerated BA. Although the effect size estimates were attenuated after covariate adjustment for demographic characteristics, socioeconomic status, lifestyle choices, and prevalent chronic diseases, these associations still remained statistically significant as shown in Table 3 (*p* < 0.05). In the fully adjusted model, participants with high CRF showed 42% lower odds of PAA (OR: 0.58; 95% CI: 0.36–0.96). Upon the above analysis, we noticed that there was no significant difference in the PAA between low and moderate CRF groups (*p* > 0.05). Therefore, in accordance with the predefined criteria for CRF impairment, we conducted a further analysis using a linear regression model with PA as a continuous variable. The results revealed that participants having impaired CRF had 1.46 units increase in their PA values compared with those without CRF impairment (*β*: 1.46; 95% CI: 1.03, 2.10; Supplementary file 4).

**Table 3:** Association between CRF and BA in different logistic models.

Characteristics	Model I		Model II		Model III	
	PAA	*p*–Value	PAA	*p*-Value	PAA	*p*-value
	OR (95% CI)		OR (95% CI)		OR (95% CI)	
CRF						
Low	ref		ref		ref	
Moderate	0.58 (0.36, 0.91)	0.026*	0.69 (0.41, 1.17)	0.2	0.71.(0.34, 1.45)	0.3
High	0.50 (0.35, 0.70)	<0.001**	0.59 (0.41, 0.86)	0.008*	0.58 (0.36, 0.96)	0.033*

* *p* < 0.05; **p < 0.001.

Model Ⅰ was not adjusted.

Model Ⅱ was adjusted for age, gender, ethnicity, marital status, education background, and poverty income ratio.

Model Ⅲ was adjusted for age, gender, ethnicity, marital status, education background, poverty income ratio, cigarette smoking, alcohol consumption, physical activity, and obesity.

### 3.3 Subgroup analyses

**Figure 2:**
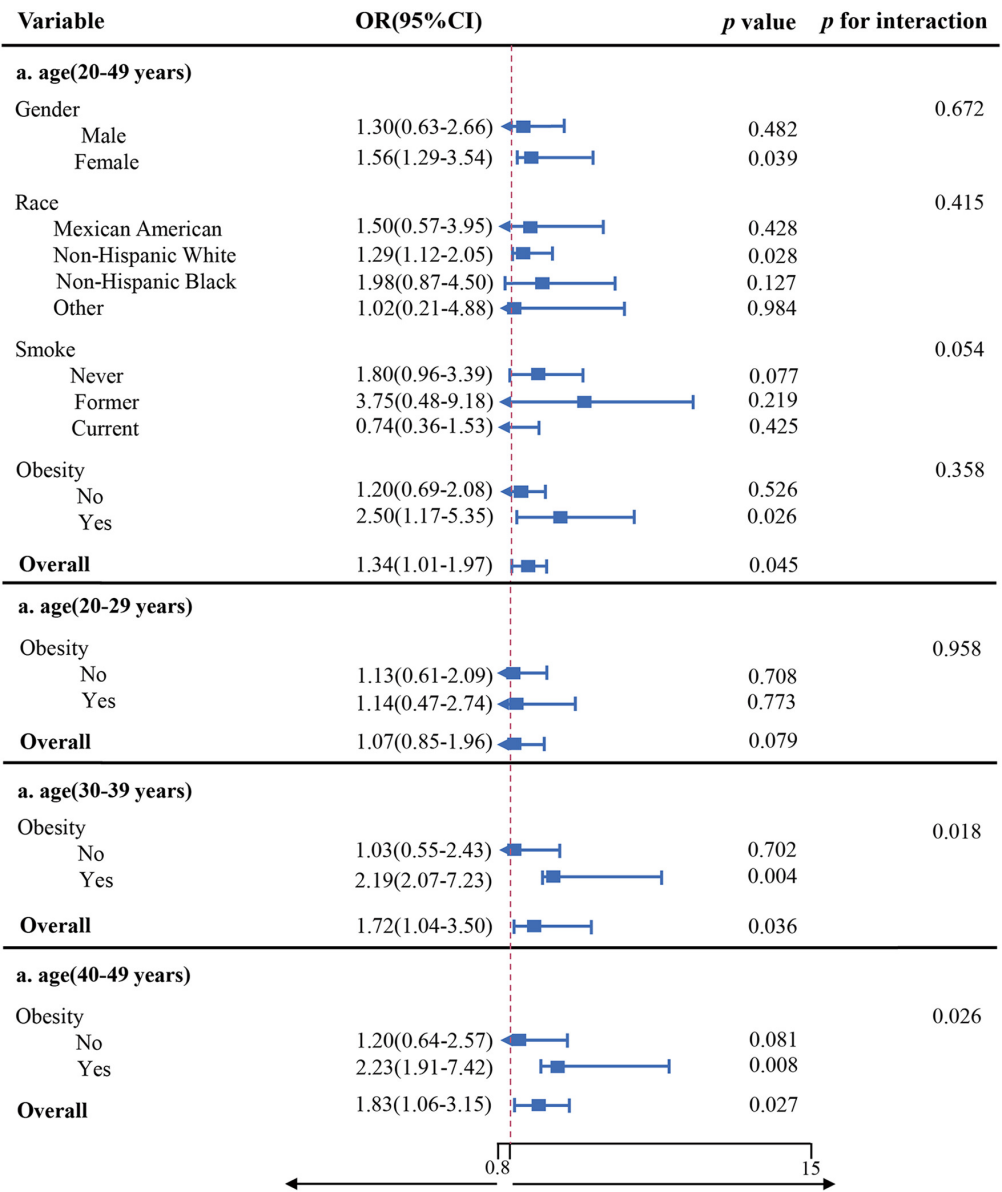
Subgroup and interaction analyses of the CRF impairment and PAA.

Weighted logistic regression analysis that adjusted for all variables presented no significant interactions between gender, ethnicity, smoke, obesity, and impaired CRF for PAA (all *p* > 0.05). In obese individuals, impaired CRF displayed a much stronger association with PAA. Subgroup analyses were repeated as we further subdivided the population according to predefined age groups, presenting a significant interaction between impaired CRF and obesity status for PAA among people over the age of 29, as shown in Figure 2 (*p* = 0.018; 0.026).

## 4 Discussion

Based on the 1999–2004 NHANES database, we exerted the first effort to reveal the relationship between the CRF status and phenotypic age among younger Americans, filling the gap in the research on CRF and BA measured by clinical parameters. Our findings suggested that good CRF is associated with a slow progression of PAA during the first half of life, which underscored its prognostic value in both individual health span trajectories and societal healthy aging processes. Moreover, a significant interaction of obesity on the relationship between CRF impairment and PAA in the population with an age over 29 years was also detected.

The single measure of CRF used to investigate its relationship with BA assessed by other methods was typically obtained during participants' midlife (45–55 years) and beyond, calling the generalizability and applicability of such an association in younger populations into question (21). Health span extension and healthy aging advancement entails not only focusing on the scope for health improvement in the elderly currently, but also attending to whether subsequent cohorts will be healthier than preceding ones (22). Regarded as the “aging but not old” generation driving future trends, youngsters are the ultimate market for antiaging therapies, and their physiological reserves are undeniably concerned with the capability of addressing the challenges posed by future aging societies. Moreover, evidence indicates that exposures to potentially preventable risks and causes of age-related diseases may stem from early in life course. And, when interventions aim to forestall accelerated aging and preempt health span losses, the schedules should better to be implemented before the fifth decade of life, considering aging is a dynamic process commonly conceptualized as “damage accrual” (23). That is to say, interventions during young adulthood may yield greater health span returns by targeting early biological aging processes. In view of this, we conducted the study focusing on the group of young participants. To some extent, it mitigated the issues more prevalent in older samples, such as selective survival, measurement biases of earlier risks or exposures, and “noise” from health ailments, therapeutic regimens, and treatment responses (24). Our finding that each step up in CRF level corresponded to a significant 29%–42% reduction in an accelerated BA risk measured by phenotypic age furnished added proof backing up CRF's active involvement in varying the intrinsic aging rates among people under 50 years’ old. This could shed light on the interpretation of the associations reported in other studies between higher CRF during youth and improved health parameters in later life, underscoring the prognostic value of CRF in age-related morbidity or mortality among younger populations.

Among those non-genetic factors, lifestyle has emerged as a potent modulator of overall health and aging (25). Compelling prior studies have demonstrated that physical activity, a crucial healthy lifestyle practice, exhibited a negative link with BA indicators measured through clinical blood chemistries to some degree (26, 27). However, most of these links have been based on self-reported questionnaires and accelerometers, with more diverse assessments and classifications of physical activity intensity, leading inconsistency, and incomparability in those research results. Moreover, individual physical limitations bring about variations in tolerance and adaptability to exercise among the same population, which may hinder such guidance from being geared to individual practical application. CRF, a unique index that integrates circulatory, pulmonary, and muscular functions into a single value, has often been conflated with physical activity, despite the two having a distinct yet related connection. Physical activity is a behavior characterized by voluntary movement generated by skeletal muscles, resulting in energy expenditure and providing a snapshot of exercise and training (28). In contrast, CRF is an objective measure of an individual's capacity to be physically active, reflecting the result of past physical activity, and can be tracked and compared across populations over time (29). Studies involving young and middle-aged adults indicated that training programs of various intensities do improve Vo_2max_ or Vo_2peak_, and increased engagement in vigorous physical activity can even enhance these parameters by up to 10% (30). This may be more instructive for individuals to find out the most suitable exercise regimen to foster a positive lifestyle cycle, with CRF and physical activity complementing each other.

In the population being over 29 years’ old, a significant association between impaired CRF and PAA among obese individuals, as represented by BMI > 30 kg/m^2^, was found. However, such an association was not evident in younger people. Previous studies have indicated that obesity status accelerates aging that used the epigenetic clock and telomere as markers in later stages of adulthood rather than in earlier periods (31, 32). The acceleration of BA may be attributable to active tissue growth or regeneration, which is particularly pronounced in adolescents and young adults during their continuous growth phase characterized by a high number of cell divisions. During this phase, it could become hard to single out the impact of increased BMI on BA, compared with normal growth-induced aging. Once the active growth period ends and the rate of BA becomes steady as a function of chronological age, the additional fat tissue can be seen as part of growth (8, 33). Therefore, the association between impaired CRF and PAA among obese individuals only primarily being detected in their middle to late adulthood does not negate the possibility of its existence at a much earlier stage of lifespan, for such an association is likely masked by confounders in younger age groups. This may also partly account for the absence of interactions between obesity and impaired CRF for PAA in the early 20 s. The cumulative adverse effects of exposure to obesity on physiological systems could be more obvious as the adaptation of individuals to metabolic stressors declines in later adulthood. Abnormal BMI rises can elevate the levels of cardiovascular risk factors through multiple pathways, including heightened oxidative stress, chronic inflammation, and metabolic and immune imbalances, imposing undue burdens on cardiopulmonary function and affecting aging process and health span in humans consequently (34). Lower values of Vo_2max_ are more frequent in individuals with a higher BMI (35). Apart from this, sympathetic discharges, muscle fibers, and blood volume also vary significantly with increasing BMI, suggesting the possibility of compromised and deconditioned cardiopulmonary efficiency among obese individuals (36). Therefore, identifying subjects with impaired CRF and taking action to mitigate the aggregating effects of obesity to decelerate the aging rate seem crucial.

The findings in the present study offered valuable evidence into the role of CRF status accounting for accelerated BA measured through phenotypic age in relatively young populations. Although data derived were from an earlier period, the association between CRF and BA relies on biological mechanisms of aging (e.g., accumulation of oxidative damage, dysregulated metabolic homeostasis) rather than era-specific lifestyle trends. Given the trans-era applicability of these biological mechanisms, these findings can still provide a theoretical foundation for contemporary antiaging interventions targeting younger populations. However, the interpretation of our results is still limited in several ways. First, as this survey is a cross-sectional study, the conclusions drawn can only provide clues for etiological research, making further longitudinal data analyses to validate the causality between CRF and PAA a necessity, particularly across developmental transitions (e.g., adolescence to young adulthood). Such investigations would complement mechanistic understanding and inform life course. Second, the analysis was reliant on the Vo_2max_ data obtained from submaximal treadmill testing rather than maximal treadmill testing in NHANES, which may have a potential influence on the final analysis due to discrete accuracies. Third, the lower diabetes prevalence in our cohort (3.5% vs. ∼8% national estimates during the same period) stemmed from excluding participants with pre-existing conditions and focusing on younger adults (20–49 of those who bear a relatively light burden of diabetes) (37), which may obscure the modulation of biological aging of CRF across dysglycemia spectra—particularly critical given the evidence that diabetes/prediabetes can affect internal aging via shared hyperglycemia-induced senescence in cells (38). Compelling evidence indicated its potential impact on aging, amplifying the low impact of CRF on all-cause mortality by almost twofold (39). The design of our study precluded detection of this mechanistic interaction. Although PhenoAge partially captured dysglycemia via glucose biomarkers (19.5% algorithm weight), it still likely underestimated the synergistic effect from combined CRF impairment and metabolic dysregulation. Finally, although our study participants were predominantly non-Hispanic White and US-centric in sampling, it is critical to distinguish between dimensions of generalizability. While derived from US mortality patterns (as in foundational studies like ACLS/VA/FIT), the core relationship between CRF and PAA operates through fundamental biological pathways conserved across populations. Thus, while external validity for population-specific effects required further verification, the internal validity for probing the role of CRF in biological aging remains robust. Collectively, this work pioneers the use of clinically accessible biomarkers to quantify biological aging in younger adults, yielding actionable insights for early interventions.

## 5 Conclusions

In this study, we found that enhancing CRF status among younger populations correlated with a deceleration in BA assessed by clinically observable characteristics, which holds valuable value for differentiating persons at risk from their peers when it comes to future age-related morbidity and mortality. In addition, obesity may exert an influence on PAA, with obese individuals being more susceptible to PAA under similar CRF conditions. Against the backdrop of accelerated global population aging, prioritizing the prevention of health span loss in younger populations has become a public health imperative. Initiating CRF interventions to slow down BA from early life may represent one of the pathways to alleviate the burden of disease and disability and improve overall health in the coming decades.

## Data Availability

Publicly available datasets were analyzed in this study. These data can be found here: The datasets analyzed in this study can be accessed on the NHANES—National Health and Nutrition Examination Survey Homepage (cdc.gov).
