# Supplementary material for: Association of cardiorespiratory fitness with phenotypic age in younger population: a study based on the NHANES database
Source: Front Sports Act Living. 2025 Jul 10;7:1503135. doi: 10.3389/fspor.2025.1503135 (PMC12286991; doi:10.3389/fspor.2025.1503135)
Supplement: Supplementary file 4 [file Table2.docx]

|  | **Model Ⅰ** | | **Model Ⅱ** | | **Model Ⅲ** | |
| --- | --- | --- | --- | --- | --- | --- |
|  | **PA** | ***p* value** | **PA** | ***p* value** | **PA** | ***p* value** |
|  | *β* (95% CI) |  | *β* (95% CI) |  | *β* (95% CI) |  |
| CVF |  |  |  |  |  |  |
| Non-impaired | ref |  | ref |  | ref |  |
| Impaired | 1.68(1.08, 2.61) | 0.023^*^ | 1.44(1.06, 2.38) | 0.031^*^ | 1.46 (1.03, 2.10) | 0.040^*^ |

Table S1 Association between CRF impairment and PA in different Logistic models

* *p＜0.05*

*** p < 0.001*

Model Ⅰ was not adjusted.

Model Ⅱ was adjusted for age, gender, ethnicity, marital status, education background, and poverty income ratio.

Model Ⅲ was adjusted for was adjusted for age, gender, ethnicity, marital status, education background, poverty income ratio, cigarette smoking, alcohol consuming, physical activity, and obesity.
